# On the identity of *Leptoxis taeniata* – a misapplied name for the threatened Painted Rocksnail (Cerithioidea, Pleuroceridae)

**DOI:** 10.3897/zookeys.697.14060

**Published:** 2017-09-14

**Authors:** Nathan V. Whelan, Paul D. Johnson, Jeffrey T. Garner, Ellen E. Strong

**Affiliations:** 1 5308 Spring ST., Warm Springs Fish Technology Center, US Fish and Wildlife Service, Warm Springs, GA 31830, USA; 2 2200 Highway 175, Alabama Aquatic Biodiversity Center, Alabama Department of Conservation and Natural Resources, Marion, AL 36756, USA; 3 350 County Road 275, Alabama Department of Conservation and Natural Resources, Florence, AL 35633, USA; 4 PO Box 37012, MRC 163, National Museum of Natural History, Smithsonian Institution, Washington, DC 20013-7012, USA

**Keywords:** Gastropoda, snails, Pleuroceridae, nomenclature, taxonomy, Mobile River Basin

## Abstract

The Painted Rocksnail, currently known as *Leptoxis taeniata*, is a federally threatened species native to the Mobile River basin in Alabama, USA. Presently restricted to four disjunct populations, the species is at considerable risk of extinction after a range decline of over 95% in the 20th century because of habitat alteration following impoundment of the Coosa River. Here, we reassess the identity and historical range of the Painted Rocksnail to improve communication and conservation efforts for the species. We determined that *L. taeniata* is a synonym of *L. picta* and that the name *L. taeniata* has been misapplied to the current concept of the Painted Rocksnail for which *L. coosaensis* is the oldest available name. *Leptoxis coosaensis* and *L. picta* are herein redescribed. After examination of historical material, we determined that records of the Painted Rocksnail outside the Coosa River drainage were misidentifications. Thus, we redefine the historical range of the Painted Rocksnail as restricted to the Coosa River and select tributaries above the Fall Line at Wetumpka, Alabama, rather than extending into the Alabama River as previously thought. *Leptoxis coosaensis* is in dire need of conservation, and management plans should take into consideration the revised historical range of the species.

## Introduction

**Figure 1.**
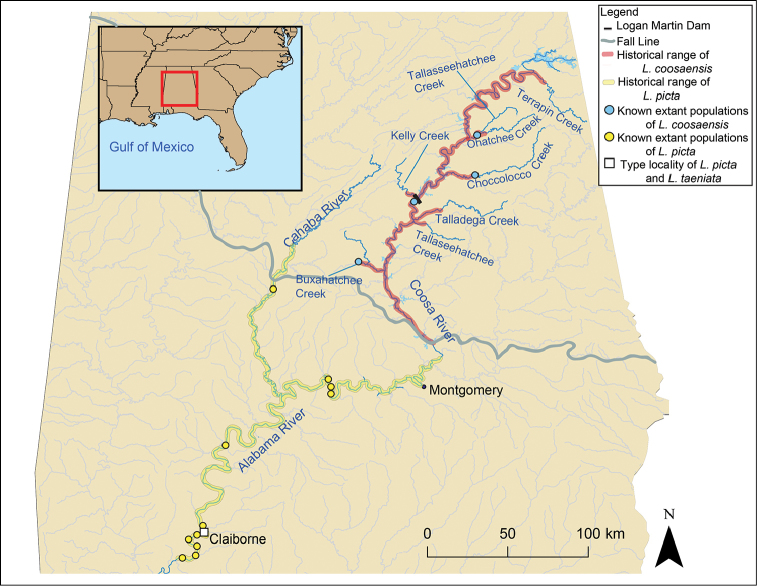
Map showing historical range of the Painted Rocksnail, *L. coosaensis*, and the Spotted Rocksnail, *L. picta*.

**Figure 2.**
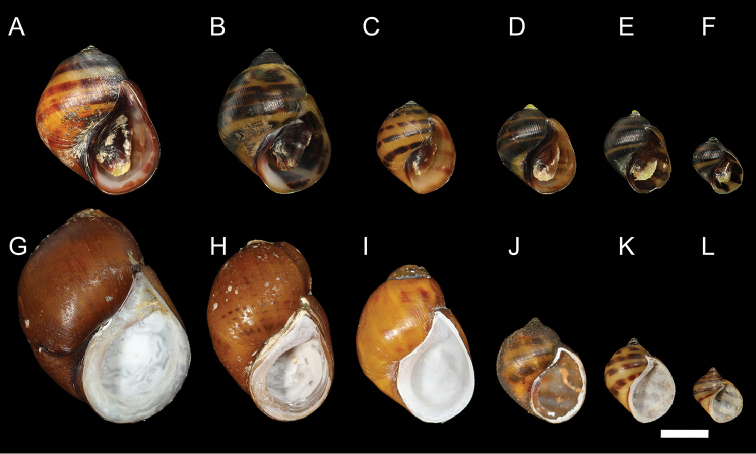
Growth series. **A–F** Adult (**A–C**) and juvenile (**D–F**) shells of the Painted Rocksnail, *Leptoxis coosaensis*. All individuals grown in captivity. **G–L** Adult (**G–I**) and juvenile (**J–L**) shells of the Spotted Rocksnail, *Leptoxis picta*. Juveniles grown in captivity. Scale bar: 4 mm.

**Figure 3.**
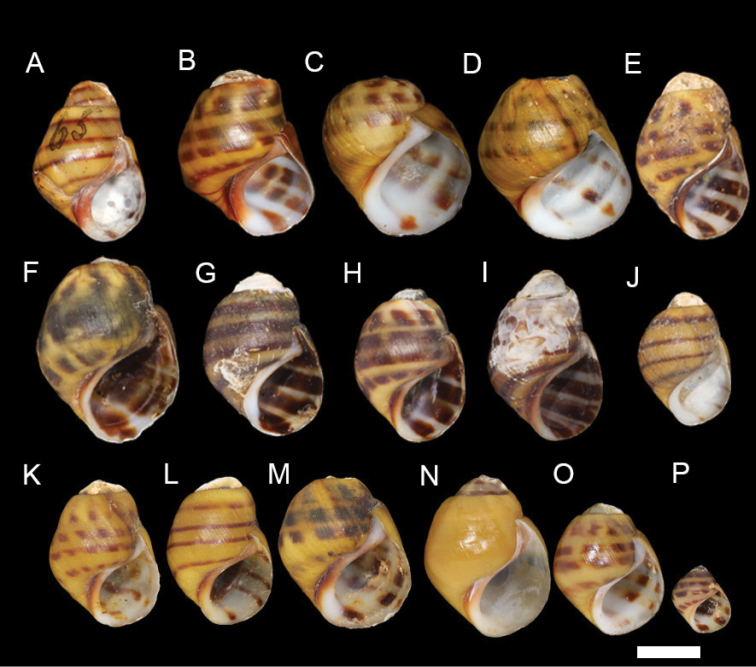
Type material of *L. coosaensis* and its synonyms and other specimens showing conchological variation seen in *L. coosaensis*. **A** USNM 121295, *Anculosa coosaensis* Lea, 1861 (lectotype) **B** UMMZ 101139, *Anculosa aldrichi* Goodrich, 1922 (holotype) **C** UMMZ 10144, *Anculosa brevispira* Goodrich, 1922 (holotype) **D** UMMZ 10145, *Anculosa choccoloccoensis*, Goodrich, 1922 (holotype) **E** USNM 1456804, *Anculosa coosaensis* Lea, 1861 (paralectotype) **F–I** USNM 121294 *Anculosa coosaensis* Lea, 1861 (paralectotypes) **J** USNM 121296 *Anculosa coosaensis* Lea, 1861 (paralectotype) **K, L** USNM 504866 "*Leptoxis taeniata*". **N–P** USNM 336408 "*Leptoxis taeniata*". Scale bar: 5 mm.

**Figure 4.**
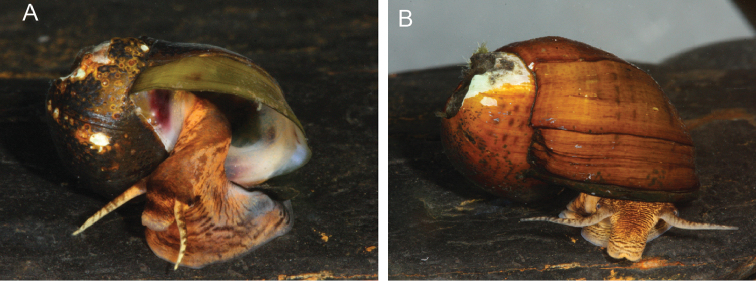
Photographs of live animals. **A** Painted Rocksnail (*L. coosaensis*) **B** Spotted Rocksnail (*L. picta*)

The Painted Rocksnail is a riverine gastropod in the family Pleuroceridae that is listed as threatened under the US Endangered Species Act (Clark 1998). It is currently restricted to four disjunct locations in the Coosa River drainage in Alabama: Choccolocco Creek, Buxahatchee Creek extending into Watson Creek, Ohatchee Creek, and Coosa River in the tailwater below Logan Martin Dam (Fig. 1). The Painted Rocksnail is characterized by a globose shell with an inflated body whorl, a large, ovate aperture, and a very low spire (Figs 2, 3). Most individuals are prominently banded, with four reddish brown spiral bands that are usually interrupted (Figs 2, 3); the head-foot is orange with mottled black patches and has a black band across the head and another across the middle of the snout (Fig. 4). Painted Rocksnails lay eggs in small clutches of approximately 3–5 eggs with limited organic and/or inorganic matter incorporated into egg casings (Whelan et al. 2015). Given its threatened status, an evaluation of the identity of the Painted Rocksnail was necessary to facilitate communication about the species, and to clarify its historical range, both of which are vital for management and recovery efforts (Hartfield 2005).

**Figure 5.**
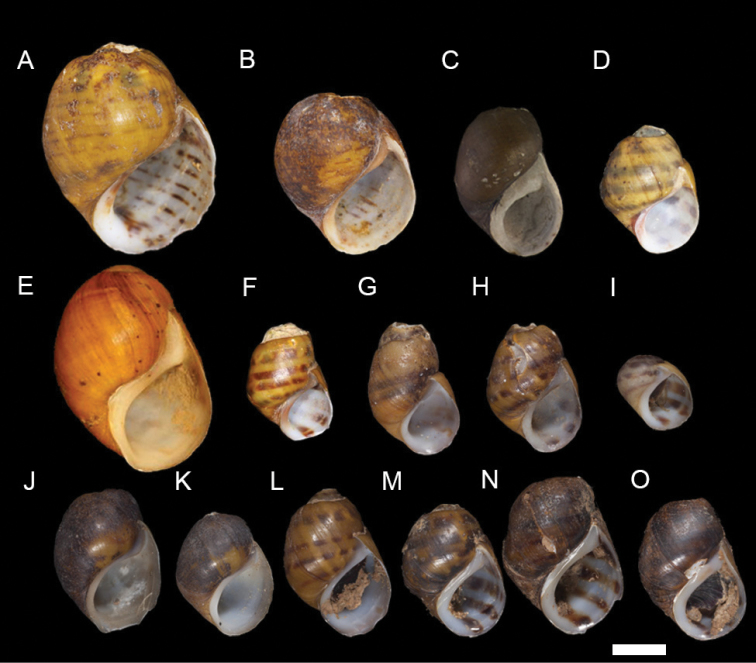
Type and topotypic material of *L. picta* and its synonym *L. taeniata*. **A, B** USNM 12074, *Anculosa picta* Conrad, 1834 (possible syntypes) **C** MCZ 294989, *Anculosa picta* Conrad, 1834 (possible syntype) **D** NHMUK uncatalogued, *Anculosa taeniata* Conrad, 1834 (possible paralectotype) **E** MCZ 294987, *Anculosa taeniata* Conrad, 1834 (paralectotype) **F** ANSP 27620, *Anculosa taeniata* Conrad, 1834 (lectotype) **G–I** ANSP 413583, *Anculosa taeniata* Conrad, 1834 (paralectotypes) **J, K** ANSP 187076, “*Leptoxis taeniata*”, collected by J.H. McLellan, unknown year (topotypic). **L–O** ANSP 65451, “*Leptoxis taeniata*”, collected by C.W. Johnson, 1894 (topotypic). Scale bar: 5mm.

The scientific name of the Painted Rocksnail is *Leptoxis taeniata* (Conrad, 1834) (Conrad 1834b), originally described under the name *Anculotus taeniatus* Conrad, 1834 from the Alabama River at Claiborne. *Anculotus* is an incorrect subsequent spelling of *Anculosa* Say, 1821, which is a junior objective synonym of *Leptoxis*. Conrad characterized *L. taeniata* as, “oval, or oblong; olivaceous, with dark green spiral bands, four on the body whorl; one whorl of the spire not eroded, often longitudinally produced” (Conrad 1834b: 63). This vague description could apply to several Mobile River drainage *Leptoxis* species including *L. picta* (Conrad, 1834) (Conrad 1834a), *L. ampla* (Anthony, 1855), and the current concept of the Painted Rocksnail. In the original description, Conrad (1834b) did not provide a figure nor did he designate a holotype or indicate a repository for the type material. Baker (1964) designated a lectotype (ANSP 27620 [as “27620a”]; Fig. 5F) from a lot of five possible syntypes in the Academy of Natural Sciences of Philadelphia (ANSP) that was labeled as “Ex auct” (i.e. “from the author”). The locality was noted simply as “Alabama”, rather than Alabama River or Alabama River at Claiborne as in the original description. Consequently, there could be some question as to whether these were syntypes and, thus, whether Baker’s designation is valid. However, as stated, the material was received from Conrad and the ANSP has type material of other taxa named by Conrad in 1834. Although the locality information is incomplete, the original label is missing, and the display label may have included only abbreviated locality information. Thus, there is insufficient evidence to overturn Baker’s lectotype designation. The collections of the Museum of Comparative Zoology at Harvard (MCZ) contain a lot of four additional paralectotypes (MCZ 294987) bearing an original A.A. Gould label stating “Anc. taeniatus Conr. Claiborne Al. from Conrad” (Fig. 5E). We also located an uncatalogued lot of four specimens containing another possible paralectotype at the Natural History Museum in London (NHMUK; *ex* Cuming collection), again labeled with the locality simply as “Alabama” (Fig. 5D). The latter lot is accompanied by an original J.G. Anthony label, annotated in his hand, “the separate one is authentic, marked so by Conrad himself, are not the others mature forms of the same?” Although one specimen is no longer conspicuously separated from the rest, we have concluded that the smallest specimen within the lot may be the specimen referred to in Anthony’s note. Although it was received from Conrad, given the ambiguity about the identity of the specimen Anthony was referring to, and the locality inconsistency, we consider it only a possible type.

Pleurocerid species display high levels of morphological variation in their shells, which can overlap between close relatives (Goodrich 1922; Whelan et al. 2015). Consequently, confidently identifying species using shells alone can be difficult, particularly for poorly localized historical material including types. As such, we carefully examined possible type material of *L. taeniata* to determine if it matches the current concept of the Painted Rocksnail. Baker’s lectotype (ANSP 27620) has a less inflated body whorl and is more elongately conical than the Painted Rocksnail. The paralectotypes in lot ANSP 413583 (formerly 27620) (Fig. 5G–I), particularly the adult specimens (Fig. 5G, H), are more clearly not representative of the Painted Rocksnail, indicating that the lectotype is a slightly atypical shell. The specimen labelled by Anthony at NHMUK (Fig. 5D) has a more narrowly ovate aperture than that seen in Painted Rocksnails. MCZ 294987, the one lot explicitly from the type locality (Fig. 5E), has a shell morphology that clearly does not conform to the current concept of the Painted Rocksnail (Figs 2, 3). Its body whorl is more narrowly conical and the aperture is more narrowly ovate, rather than broadly ovate in large adults. Painted Rocksnails also usually have more impressed sutures. Overall, the possible type material of *L. taeniata* (Fig. 5) does not conform with the current concept of the Painted Rocksnail.

In addition to examining type and historical material (Fig. 5), we evaluated pleurocerid collections from the Alabama River made in the last 30 years to help determine the identity and range of the Painted Rocksnail. No modern survey has recovered the Painted Rocksnail from any location in the Alabama River (Garner et al. 2011). These surveys included over 190 hours of dive time since 1990 in the Alabama River (JT Garner, unpubl. data). Furthermore, examination of historical museum collections has failed to produce a single lot from the Alabama River that corresponds to the current concept of the Painted Rocksnail. Lots labeled as “*L. taeniata*” from the Alabama River are misidentified specimens, usually of *L. picta* (e.g. UF 82371, ANSP 65451 Fig. 5L-O; also see photographs uploaded to FigShare, https://doi.org/10.6084/m9.figshare.5084272.v1); lots identified as “*L. taeniata*” from the Cahaba River drainage are also misidentified, usually of specimens of *L. ampla* (e.g. UF 81652, USNM 519194; see photographs uploaded to FigShare, https://doi.org/10.6084/m9.figshare.5084272.v1). Currently, the Alabama River near Claiborne hosts healthy populations of other pleurocerids including *L. picta*, *Pleurocera prasinata* (Conrad, 1834) (Conrad 1834b), and multiple *Elimia* species (Garner et al. 2011). Therefore, we doubt that the apparent failure to collect the Painted Rocksnail from its ostensive type locality for over 150 years reflects extirpation of the species at that site.

We also considered the historical range of the Painted Rocksnail as presently understood from a biogeographic perspective. The Alabama River is exclusively below the Fall Line, a major physiographic break that separates the Gulf Coastal Plain from the Appalachian Highlands (Renner 1927). No other *Leptoxis* species from the Coosa River above the fall line has a historical range that extends into the Alabama River or Coastal Plain physiographic region. *Leptoxis ampla*, a Cahaba River endemic and the sister species to the Painted Rocksnail (Whelan et al. 2015), is also found only above the Fall Line. Therefore, if the Painted Rocksnail was historically found in both the Alabama River and the Coosa River above Wetumpka, the species would represent a significant departure from distribution patterns seen among Mobile River basin *Leptoxis* species.

Despite examining records at seven major natural history collections [ANSP, MCZ, NHMUK, University of Michigan Museum of Zoology (UMMZ), National Museum of Natural History (USNM), North Carolina Museum of Natural Sciences (NCMNS), and Florida Museum of Natural History (UF)], we have not located a single lot from the Alabama River that could conclusively be identified as the Painted Rocksnail. After careful examination of historical collections and consideration of both contemporary surveys and broad biogeographic patterns, we conclude that all possible type material of *L. taeniata* more closely resembles *L. picta* than the current concept of the Painted Rocksnail.

*Leptoxis picta* was described from the same location as *L. taeniata*. The original description of *L. picta* is sufficiently vague that it could be applied to multiple Mobile River drainage *Leptoxis* species. *Leptoxis picta* was described as, “Shell sub-oval, shoulder obtusely rounded; aperture ovate, large; columella callous above; epidermis olive, with numerous quadranglular small spots disposed in revolving lines, strongly marked in the aperture” (Conrad 1834a: 342–343). To differentiate the two species, Conrad (1834b) noted that *L. picta* often had pigmentation spots and that *L. taeniata* had dark green bands, but both patterns have been documented in both species (Figs 2, 3, 5). Further, *Leptoxis picta* was described as inhabiting pebble bars, whereas *L. taeniata* was observed to inhabit friable calcareous banks and siliceous breccias (Conrad 1834b). We question whether any pleurocerid species could be reliably distinguished based on minor differences in habitat preference as we have often observed individuals of the same *Leptoxis* species to inhabit many different microhabitats (i.e. near the banks and in the main current, both pebble and bedrock substrates; Whelan et al. pers. obs.).

## Results

Taking all the above into consideration, we have concluded that the type material of *L. taeniata* and *L. picta* represents the same taxonomical species and that the two are synonyms. *Leptoxis picta* was described four months prior to *L. taeniata* and thus has priority under Article 23 of the International Code of Zoological Nomenclature (ICZN). Nevertheless, the current concept of the Painted Rocksnail represents a unique monophyletic clade in phylogenetic analyses of *Leptoxis* species, and possesses body coloration patterns (Fig. 4) and egg laying behavior different from that of *L. picta* (Whelan et al. 2015). Misapplication of the name *Leptoxis taeniata* to specimens from the Coosa River apparently became widespread after the publication of Tryon (1873), when *L. coosaensis* (Lea, 1861) was synonymized with *L. taeniata*. The name *taeniata* does not meet requirements of Art. 23.9.1 of the ICZN that prevailing usage must be maintained because the name has not been used as valid in 25 or more publications in the last 50 years (Burch 1982; Burch and Tottenham 1980; Dillon and Lydeard 1998; Holznagel and Lydeard 2000; Johnson et al. 2013; Lee et al. 2006; Lydeard et al. 1997; Lydeard et al. 1998; Strong and Köhler 2009; Tolley-Jordan et al. 2015; Whelan 2016; Whelan et al. 2015). In such instances, one possible course of action would be conservation of prevailing usage by designation of a neotype under Art. 75.6, which would require a request to the Commission to use its plenary powers to set aside any existing name-bearing types and select a neotype. However, the name *L. taeniata* has long been associated with an incorrect historical distribution for the Painted Rocksnail and further use of the name could perpetuate this error and create confusion for future management plans. Thus, rather than maintain prevailing usage, we here prefer to recognize the oldest available name for the Painted Rocksnail, which we have determined to be *Leptoxis coosaensis* (Lea, 1861).

## Systematics

### Leptoxis coosaensis

Taxon classificationAnimaliaNeotaenioglossaPleuroceridae

(Lea, 1861)

Anculosa coosaensis Lea, 1861: 54; 1863a: 257–258, pl. 35, fig. 65; 1863b: 79–80, pl. 35, fig. 65. Lectotype USNM 121295 (Graf, 2001); paralectotypes USNM 1456804 (1 spm), USNM 121294 (4 spms) and USNM 121296 (1 spm) *leg.* Showalter. “Coosa River, Alabama.”
Anculosa aldrichi Goodrich, 1922: 31, pl. 1, figs. 1, 2. Holotype UMMZ 10139, by original designation; paratype, MCZ 169987 (1 spm). “Coosa River, near mouth of Yellowleaf [*sic*, Yellow Leaf] Creek, Chilton County, Alabama.”
Anculosa brevispira Goodrich, 1922: 35, pl. 1, fig. 6. Holotype UMMZ 10144, by original designation; paratype MCZ 169991 (1 spm). “Fort William Shoals, Coosa River, Talladega County, Alabama”; possible paratypes UF 18202 (7 spms). “Coosa River”.
Anculosa choccoloccoensis Goodrich, 1922: 34, pl. 1, fig. 7. Holotype UMMZ 10145, by original designation; paratype MCZ 169989 (1 spm). “Choccolocco Creek at Jackson Shoals, Talladega County, Alabama.”
Anculosa taeniata lucida Goodrich, 1944: 42. Type material not located, potentially lost. “Coosa [River] tributaries.”
Other references: Anculosa coosaensis —Lea 1863: 257-258, pl. 35, fig. 65; Goodrich 1922: 28, pl. 1, figs. 13, 14.
Leptoxis taeniata —Haldeman 1848: 3, pl. 3 figs. 71–72; Burch and Tottenham 1980: 156, figs. 484–486; Burch 1982: 43, figs. 484–486; Lydeard et al. 1997: 117–128; Lydeard et al. 1998: 183–193; Dillon and Lydeard 1998: 113–121, fig. 2; Holznagel and Lydeard 2000: 233–257; Lee et al. 2006: 314–317; Strong and Köhler 2009: 483–502; Tolley-Jordan et al. 2015: 235–249; Whelan et al. 2015: 85–95, fig. 4; Whelan 2016: 221–226. [Not L. taeniata of Conrad]
Anculosa taeniata —Tryon 1873: 408–409, figs. 813–815. [Not L. taeniata of Conrad]
Anculotus taeniatus —Reeve 1860: pl. 6, fig. 50. [Not L. taeniata of Conrad]

#### Other material examined.

UMMZ 10144, Coosa River at Fort Williams Shoals, Talladega County, Alabama (~33.1477°N, 86.4831°W); UMMZ 10139, Coosa River near mouth of Yellow Leaf Creek, Chilton County, Alabama (~32.9566°N, 86.5177°W); UMMZ 10145, Choccolocco Creek at Jackson Shoals, Talladega County, Alabama (~33.5450°N, 86.0896°W); MCZ 169987, Coosa River near mouth of Yellow Leaf Creek, Chilton County, Alabama (~32.9566°N, 86.5177°W); MCZ 169989, Choccolocco Creek at Jackson Shoals, Talladega County, Alabama (~33.5450°N, 86.0896°W); USNM 12068, Coosa River, Alabama; USNM 321181, Duncan’s Riffle Coosa River, Chilton County (~32.8057°N, 86.4450°W; USNM 321862, Higgin’s Ferry Coosa River, Chilton County, Alabama (~32.8056°N, 86.4448°W); UF 82401, Peckerwood Shoals, Talladega County, Alabama (~33.1176°N, 86.4728°W); UF 416903, Talladega Creek near Nottingham, Talladega County, Alabama (~33.3614°N, 86.2237°W); UF 82358, Coosa River at Fort Williams Shoals, Talladega County, Alabama (~33.1477°N, 86.4831°W); UF 81660, Coosa River at Fort Williams Shoals, Talladega County, Alabama (~33.1477°N, 86.4831°W); UF 82419, Coosa River at Butting Ram Shoals, Coosa County, Alabama (~32.9414°N 86.5159°W); UF 413800, Coosa River at Lonigan Shoals, St. Clair County, Alabama (~33.7627°N, 86.0447°W); UF 81085, Choccolocco Creek at Jackson Shoals, Alabama (33.5450°N, 86.0896°W); UF 18202, Coosa River; UF 416913, Coosa River at Wetumpka, Elmore County, Alabama (~32.5396°N, 86.2056°W); UF 416911, Coosa River at Wetumpka, Elmore County, Alabama (~32.5396°N, 86.2056°W); UF 416908, Waxahatchee Creek 4 mi above mouth, Shelby County, Alabama (~33.0335°N, 86.5787°W); UF 416920, Kellys [*sic*, Kelly] Creek 2 mi above mouth, St. Clair County, Alabama (~33.4348°N, 86.3539°W); UF 416897, Tallasahatchee [*sic*, Tallaseehatchee] Creek 2-3 mi east of Childersburg, Talladega County, Alabama (~33.2841°N, 86.3098°W); UF 416917, Beeswax Creek, Shelby County, Alabama; NCSM 59354, Terrapin Creek 7 mi south of Centre, Cherokee County, Alabama (~34.0639°N, 85.6146°W); NCSM 59359, Tallahatchie [*sic*, Tallasseehatchee] Creek, Calhoun County, Alabama (33.7785°N, 85.9908°W); NCSM 59358, Ohatchee Creek, Calhoun County, Alabama (33.7801°N, 85.9972°W); USNM 504866, Coosa River, near Wilsonville, Shelby County, Alabama (~33.2162°N, 86.4645°W); USNM 504867, Coosa River 1 mi from Wilsonville, Shelby County, Alabama (~33.2162°N, 86.4645°W); USNM 336408, Coosa River at The Bar, Chilton County, Alabama (~32.7976°N, 86.4348°W); USNM 1437765, Coosa River, Shelby County, Alabama (33.3744°N, 86.3550°W); USNM 1249597, Choccolocco Creek, Talladega County, Alabama (33.5445°N, 86.0414°W); USNM 1249600, Buxahatchee Creek, Shelby County, Alabama (33.0727°N, 86.6775°W); USNM 1437762, Ohatchee Creek, Calhoun County, Alabama (33.7795°N, 86.0002°W); USNM 1437763, Ohatchee Creek, Calhoun County, Alabama (33.7795°N, 86.0002°W); USNM 1437764, Ohatchee Creek, Calhoun County, Alabama (33.7795°N, 86.0002°W).

#### Diagnosis.

Shell ovate, two to four whorls, spire often reduced to obsolete but sometimes elevated with obtuse apex. Aperture large, ovate, at least half the height of body whorl. Reddish brown spiral bands typically present, usually four in number, almost always interrupted. Columella often purple. Head-foot and mantle pigmented orange, mottled with black, with one transverse black band across middle of snout and one transverse black band across middle of head. Clutches small (<6 eggs), with minimal organic and/or inorganic matter incorporated into external casings.

#### Historical distribution.

Coosa River above the Fall Line from Wetumpka, Alabama, upstream to the confluence of Terrapin Creek and the Coosa River in Cherokee County, Alabama. Some large Coosa River tributaries including Choccolocco, Buxahatchee, Talladega, and Terrapin creeks.

#### Current distribution.

Four disjunct populations: Choccolocco Creek, Talladega County, Alabama; Buxahatchee and Watson creeks, Shelby County, Alabama; Ohatchee Creek, Calhoun County, Alabama; Logan Martin Dam tailwaters of the Coosa River, Shelby-Talladega counties, Alabama.

#### Remarks.

USNM 121295 (Fig. 3A) originates from the Lea collection, is from the published *L. coosaensis* type locality, *leg.* Showalter, and is the shell figured by Lea (1863), with the number 65 inked onto the apertural aspect of the body whorl corresponding to the figure number. Lea (1863) indicated that he had six specimens, yet there are seven specimens distributed among the three simultaneously accessioned lots now registered as USNM 121294 (Fig. 3F-I), USNM 1456804 (see photographs on FigShare, https://doi.org/10.6084/m9.figshare.5084272.v1), and USNM 121296 (Fig. 3J). Consequently, either the specimen count in Lea (1863) was in error, or one of the included shells was acquired subsequently and has no type status.

Lea (1861) described *Anculosa coosaensis* from the Coosa River and provided a brief description in Latin. In a subsequent extended description in English, Lea (1863) characterized the species as, “smooth, obtusely conical, thick, dark horn-color, very much banded; spire elevated, obtuse at the apex; sutures very much impressed; whorls four, very much constricted below the sutures, the last large; aperture rounded, white, much banded within; columella thickened, incurved, dark purple; outer lip acute and expanded.” In the remarks, he commented that the aperture is more than half the length of the shell and that the bands may be interrupted. This description, as well as the type material (Fig. 3F–J) match the current concept of the Painted Rocksnail (Fig. 2). Although formerly considered a synonym of *L. taeniata*, *L. coosaensis* is the oldest available name for the Painted Rocksnail.

We have been unable to locate type material of *Anculosa taeniata lucida* Goodrich, 1944. No holotype was designated, nor was a figure provided, but based on the original description and the type locality of tributaries of the Coosa River, we conclude that this entity does not merit recognition at the subspecies level and synonymize it with *L. coosaensis*.

### Leptoxis picta

Taxon classificationAnimaliaNeotaenioglossaPleuroceridae

(Conrad, 1834)

Anculosa picta Conrad, 1834a: 343, pl. 1, fig. 16. Possible syntype MCZ 294989 (4 spms); possible syntypes USNM 12074 (2 spms). “Alabama River” [near Claiborne].
Anculosa taeniata Conrad, 1834b: 63. Lectotype ANSP 27620 (Baker, 1964; as “27620a”); paralectotypes ANSP 413583 (3 spms; formerly 27620); paralectotypes MCZ 294987 (4 spms); possible paralectotype NHMUK uncatalogued (1 spm). “Alabama River at Claiborne.”
Other references: Leptoxis picta —Haldeman 1848: 3, figs. 74–80; Burch and Tottenham 1980: 154, fig. 476; Burch 1982: 42, fig. 476; Lydeard et al. 1997: 117–128; Dillon and Lydeard 1998: 113–121, fig. 2; Holznagel and Lydeard 2000: 233–257; Lee et al. 2006: 314–317; Strong and Köhler 2009: 483–502; Tolley-Jordan et al. 2015: 235–249; Whelan et al. 2015: 85–95, fig. 4.
Anculosa picta —Tryon 1873: 415–417, figs. 829–830; Goodrich 1922: 14-15, figs. 34, 35;

#### Other material examined.

ANSP 120760, Alabama River, Alabama; ANSP 85033, Cahaba River, Alabama; ANSP 163024, Cahaba River, Lilly Shoals, Bibb County, Alabama (~33.1552°N, 87.0365°W); ANSP 65451, Alabama River at Claiborne, Monroe County, Alabama (31.5512°N, 87.5142°W); ANSP 187076, Alabama River at Claiborne, Monroe County, Alabama (31.5512°N, 87.5142°W); UF 81414, Alabama River at Selma, Dallas County, Alabama (~32.4049°N, 87.0190°W); UF 82371, Alabama River, Alabama; UMMZ 10175, Alabama River at Selma, Dallas County, Alabama (~32.4049°N, 87.0190°W); UMMZ39983, Alabama River at Selma, Dallas County, Alabama (~32.4049°N, 87.0190°W); UMMZ 57813, Cahaba River, 19.3 KM W of Selma, Dallas County, Alabama; USNM 507433, Alabama River at Claiborne, Monroe County, Alabama (31.5512°N, 87.5142°W) ; USNM 525014, Alabama River at Claiborne, Monroe County, Alabama (31.5512°N, 87.5142°W) ; USNM 121232, Alabama River at Selma, Dallas County, Alabama (~32.4049°N, 87.0190°W); USNM 519212, Alabama River at Selma, Dallas County, Alabama (~32.4049°N, 87.0190°W); USNM 1437744, Alabama River downstream of Benton boat ramp, Lowndes-Autauga Counties, Alabama (32.3214°N, 86.8215°W); USNM 1437745, Alabama River downstream of Benton boat ramp, Lowndes-Autauga Counties, Alabama (32.3214°N, 86.8215°W); USNM 1437749, Alabama River downstream of Benton boat ramp, Dallas-Autauga Counties, Alabama (32.3226°N, 86.8220°W); USNM 1437746, Alabama River at river mile 231.5, Dallas-Autauga Counties, Alabama (32.3413°N, 86.8159°W); USNM 1437750, Alabama River at river mile 70.5, Monroe County, Alabama (31.5914°N, 87.5415°W); USNM 1437758, Alabama River at river mile 223.7, Dallas-Autauga Counties, Alabama (32.4299°N, 86.8308°W); USNM 1437747, Alabama River at river mile 224.7, Dallas-Autauga Counties, Alabama (32.4210°N, 86.8337°W); USNM 1437748, Alabama River at river mile 226.5, Dallas-Autauga Counties (32.4064°N, 86.8463°W); USNM 1437759, Alabama River at river mile 227.0, Dallas-Autauga Counties, Alabama (32.3941°N, 96.8375°W); USNM 1437753, Alabama River at river mile 46, Clarke-Monroe Counties, Alabama (31.4274°N, 87.6452°W); USNM 1437754, Alabama River at river mile 51.5, Clarke-Monroe Counties, Alabama (31.4372°N, 87.5716°W); USNM 1437756, Alabama River at river mile 54.6, Clarke-Monroe Counties, Alabama (31.4734°N, 87.5620°W); USNM 1437761, Alabama River at river mile 58.0, Clarke-Monroe Counties, Alabama (31.5051°N, 87.6125°W); USNM 1437755, Alabama River at river mile 59.7, Clarke-Monroe Counties, Alabama (31.5196°N, 87.6205°W); USNM 1437760, Alabama River at river mile 64.3, Monroe County, Alabama (31.5559°N, 87.5611°W); USNM 1437757, Alabama River at river mile 75.0, Monroe County, Alabama (31.5898°N, 87.5391°W); USNM 1437752, Alabama River at river mile 75.8, Monroe County, Alabama (31.5923°N, 87.5407°W); USNM 1437743, Alabama River at river mile 128.6, Wilcox County, Alabama (32.0409°N, 87.4118°W); USNM 1437744, Alabama River at river mile 233.0, Lowndes-Autauga Counties, Alabama (32.3214°N, 86.8215°W); USNM 1437751, Alabama River 2.4 KM downstream of US Highway 84 bridge, Monroe County, Alabama (31.5455°N, 87.5367°W).

#### Diagnosis.

Shell globose, larger shells elongately globose, two to three whorls, spire reduced to obsolete. Reddish brown spiral bands typically present on smaller shells, often faded on larger shells, usually four in number, often interrupted. Head-foot and mantle pigmented orange, mottled with black. Egg clutches spiral, 10-11 eggs per clutch on average, with minimal organic and/or inorganic matter incorporated into external casings.

#### Historical distribution.

Alabama River from Claiborne, Alabama, upstream to mouth of Coosa River. Coosa River below Wetumpka. Goodrich (1922) reported *L. picta* from as far upstream as bars of the Coosa River below Wetumpka; although we have not examined any lots of *L. picta* from the Coosa River, we consider this record reliable as it is below the Fall Line. In the Cahaba River, from its confluence with the Alabama River upstream to Lily Shoals in Bibb County, Alabama.

#### Current distribution.

Disjunct populations in the Alabama River from river mile 46.0 in Monroe-Clarke counties, upstream to approximately river mile 231.5, near the Lowndes/Dallas county line. One recently reintroduced population in the Cahaba River at Centreville, Bibb County, Alabama (P.D. Johnson *unpublished data*.)

#### Remarks.

Baker (1964) doubtfully listed ANSP “120960a?” (*sic*, error for 120760a; now ANSP 120760) as the possible “TOM” of *L. picta* from among a lot of 16 specimens. Baker stated “TOM” as meaning, “type because only one example was included in the original description, or was indicated by only one set of dimensions (of course the first) or by reference to a (cited) illustration(s) of only one shell, in the definition proper, exclusive of additional remarks.” He considered use of this abbreviation as a “type by subsequent selection” (Baker 1963: 191). Consequently, his use of the abbreviation “TOM” could be a valid lectotype designation under certain circumstances. However, as Baker placed a question mark after the catalogue number indicating uncertainty that the specimen selected was the type by original measurement, he did not unambiguously select a specimen to act as the name bearing type as required by Art. 74.5 (ICZN) and hence this does not constitute a valid lectotype designation. Furthermore, there is no evidence that the lot has any type status; it originated from the Wheatley collection via the University of Pennsylvania and there is no evidence on the labels or in the original ANSP ledger that the lot was obtained from Conrad. It is possible that Baker knew that Wheatley received material from Conrad, but the original label is not in Conrad’s handwriting (G. Rosenberg, pers. comm.). Despite Conrad stating that the type material had been deposited in the ANSP, we have been unable to locate any other possible type material during several searches of the collections. USNM 12074 (Fig. 5A, B) and MCZ 294989 (Fig. 5C) both resemble the shell figured by Conrad and have labels indicating they were received from Conrad. However, both lots are accompanied by labels bearing the less-specific locality Alabama, rather than Alabama River or Alabama River at Claiborne. Moreover, as mentioned, the possible syntypes were not found in ANSP, the stated repository of the types. Consequently, it is possible that neither MCZ 294989 nor USNM 12074 are syntypical and so we refrain from designating a lectotype.

Tryon (1873) considered both *Leptoxis foremani* (Lea, 1843) and *L. flammata* (Lea, 1843) to be synonyms of *L. picta*. Burch and Tottenham (1980) restored *L. foremani* to species status, but retained *L. flammata* as a synonym of *L. picta*. Both *L. picta* and *L. foremani* are reciprocally monophyletic and valid species (Whelan et al. 2015). However, based on shell morphology we here consider *L. flammata* and *L. foremani* to be synonyms. As both were described concurrently (Lea 1843), we here take the right of First Reviser (ICZN Art. 24.2) and establish the priority of *L. foremani* over *L. flammata*, making *L. flammata* a subjective junior synonym of *L. foremani*. *Leptoxis zebra* (Anthony, 1860) was also considered by Tryon (1873) to be a synonym of *L. picta*, but the type material (MCZ 161794, see shells photographs on FigShare, https://doi.org/10.6084/m9.figshare.5084272.v1) resembles *L. foremani*. Consequently, we here consider *L. zebra* also to be a junior synonym of *L. foremani*.

## Discussion

Today, the Painted Rocksnail, i.e. *Leptoxis coosaensis*, is mostly restricted to Coosa River tributaries (Fig. 1). In tributary habitats, pleurocerids are generally smaller than main stem conspecifics. Consequently, modern specimens of wild caught individuals typically are smaller and have more eroded spires than those seen in types and some historical material. Juveniles grown in captivity with uneroded spires have four extremely compressed whorls (Fig. 2; Whelan et al. 2015). We failed to find any specimens with costae and consider the specimen with prominent costae figured in Burch and Tottenham (1980: fig. 486) as the Painted Rocksnail to be a probable misidentification, possibly of *L. showalterii*. Pleurocerids are notoriously difficult to identify, and similarities in shell morphology of *L. picta* and *L. coosaensis*, particularly of the juveniles, undoubtedly contributed to the confusion in application of an incorrect scientific name to the Painted Rocksnail for nearly 150 years (Tryon 1873).

*Leptoxis coosaensis* is listed as threatened under the Endangered Species Act as “*L. taeniata*” (Clark 1998). The species is currently restricted to four disjunct populations in the Coosa River drainage in eastern Alabama. Those in Choccolocco Creek, Ohatchee Creek, and Buxahatchee and Watson creeks appear stable, while the status of the population in the Logan Martin Dam tailwaters is unclear because of low abundance and/or difficulties associated with sampling at this location (i.e. depths that require diving in high flow and poor visibility).

Listing of this species was based, in part, on the misperception that the historical distribution included a long stretch of the Alabama River from which it had been extirpated during the 20th century (Clark 1998). Furthermore, many museum lots identified as “*L. taeniata*” represent taxonomical species different from the Painted Rocksnail, typically of *L. picta* or *L. ampla*. The latter is endemic to the Cahaba River drainage above the Fall Line in Alabama; consequently, these misidentifications have resulted in erroneous reports that the Painted Rocksnail was historically present in the Cahaba River (Burch and Tottenham 1980; Goodrich 1922; Mirachi et al. 2004). Conversely, records of *L. ampla* in the Coosa River drainage are misidentifications, typically of *L. coosaensis*. In light of our reanalysis, the historical range of *L. coosaensis* is here revised to have been restricted to the Coosa River and its tributaries, which is a considerably smaller historical range for the Painted Rocksnail than previously believed (Clark 1998). Nevertheless, the current range reduction of 90% from historical occupancy given by Clark (1998) appears to have been conservative.

The historical range of *L. coosaensis* just in the Coosa River proper is a distance of approximately 317 km (Fig. 1). Since *L. coosaensis* is now known to inhabit less than 10 km of the main stem Coosa River, its range has declined over 95% in that river alone. In addition, *Leptoxis coosaensis* is believed to be extirpated from four of the eight Coosa River tributaries from which it was known. As such, even with a redefined and reduced historical range, this species is in obvious need of continued protection. Management efforts for pleurocerid snails in the Mobile River basin have focused on habitat improvement and captive propagation and reintroduction. Reintroduction should never occur outside a species’ historical range, which is one reason why clarifying the range of *L. coosaensis* is so important. Recovery efforts should include a review of historic tributaries that once supported *L. coosaensis* to determine if any sites are appropriate for reintroduction. As with most listed mollusks, establishment of additional populations contributes to species recovery, and is necessary for the possible delisting of the species (Hartfield 2005).

## Supplementary Material

XML Treatment for Leptoxis coosaensis

XML Treatment for Leptoxis picta
